# The predictive value of the triglyceride—glucose index for cardiovascular events in patients with coronary chronic total occlusion

**DOI:** 10.1186/s12933-022-01588-4

**Published:** 2022-08-08

**Authors:** Yingkai Li, Songyuan He, Zheng Wu, Wenzheng Li, Wen Jian, Zichao Cheng, Cong Wang, Yuchen Shi, Jinghua Liu

**Affiliations:** Center for Coronary Artery Disease (CCAD), Beijing Anzhen Hospital, Capital Medical University, Beijing Institute of Heart, Lung, and Blood Vessel Diseases, Beijing, 100029 China

**Keywords:** Triglyceride-glucose index, Chronic total occlusion, Cardiovascular events

## Abstract

**Background:**

Chronic total occlusion (CTO) of the coronary artery is a difficult problem in clinical practice. The triglyceride**–**glucose (TyG) index is an effective risk predictor of cardiovascular risk. However, the relationship between the TyG index and the prognosis of CTO patients remains unstudied. Thus, the present study aimed to investigate the relationship between the TyG index and cardiovascular risk in CTO patients.

**Methods:**

This was a single-centre, retrospective cohort study. We retrospectively enrolled 652 patients with CTO lesions diagnosed by angiography and who underwent revascularization through PCI. Patients were routinely followed up for 24 months unless meeting the endpoint. The primary endpoint was the composite of all-cause death, nonfatal myocardial infarction, unplanned revascularization, and nonfatal ischaemic stroke. To test the association of the TyG index with cardiovascular risk, the categorized TyG index and Cox proportional hazards regression models were utilized.

**Results:**

A total of 652 patients were enrolled in the final analysis (male: 83.7%, age: 58.2 ± 10.49 years). The average TyG index was 8.8 ± 0.57. CTO PCIs were procedurally successfully completed in 503 (77.15%) patients. During the follow-up period of 22.8 ± 3.84 months, 73 (11.19%) major adverse cardiovascular and cerebral events (MACCEs) occurred. When fully adjusted, there was a 2.09-fold risk for MACCEs among patients with the highest TyG index compared with those with the lowest TyG index [T2 vs. T1: hazard ratio (HR) 1.24, 95% confidence interval (CI) 0.65–2.38, *P* = 0.057; T3 vs. T1: HR 2.09, 95% CI 1.14–3.86, *P* = 0.018; *P* for trend = 0.036]. The restricted cubic spline (RCS) analysis showed that the HR for MACCEs increased as the TyG index increased over 8.71 [HR per standard deviation (SD) 1.740, 95% CI 1.23–2.46, *P* = 0.002]. The risk of MACCEs increased with increasing tertiles of TyG index in successful CTO PCI patients and nondiabetes mellitus (DM) patients (*P* < 0.05) but not in patients with failed CTO PCI and DM patients.

**Conclusion:**

The study revealed that the TyG index had significant relevance to cardiovascular risk in CTO patients and suggests that the TyG index is feasible for predicting cardiovascular risk in CTO patients.

## Background

CTO of the coronary artery is difficult to address in clinical practice. With the update of clinical guidelines and the innovation of technology and instruments, the success rate of CTO PCI has been increased to 70%–95.9% [1, 2]. Successful PCI treatment could alleviate angina symptoms, improve quality of life and decrease cardiovascular events [3, 4]. However, there is still a 10.4% 10-year cardiac mortality rate in patients who received successful PCI for CTO lesions [4]. Thus, it is necessary to explore a target that can identify high-risk patients with cardiovascular events after CTO PCI to prescribe more active treatment to reduce events.

Insulin resistance (IR) is considered to be closely associated with cardiovascular risk. Studies have shown that the TyG index is reliable in reflecting IR status [5]. TyG index, which only needs to acquire the TG and fasting blood glucose levels of patients, has become an attractive choice for reflecting IR levels because of its high availability and low cost. Studies have shown a high correlation between the TyG index and previous standard IR indices, including the hyper insulinemic-euglycemic glucose clamp and HOMA-IR [6].

In some aspects, TyG is even more effective than the HOMA-IR model, especially in predicting metabolic syndrome, and arterial stiffness [7, 8]. And several studies have demonstrated that the TyG index can predict myocardial infarction, stroke or other adverse cardiovascular events [9–11]. However, no studies have investigated the relationship between the TyG index and cardiovascular events in CTO patients. Thus, we aimed to explore whether the TyG index could predict adverse cardiovascular events in CTO patients.

## Methods

### Study population

This was a single-centre, retrospective cohort study. A total of 738 patients who met the inclusion criteria were included. 69 patients were excluded due to incomplete key variables (n = 22) and the exclusion criteria (n = 47). Finally, 669 patients were enrolled in the final follow-up. The inclusion criteria consisted of age > 18 years, and diagnosis of CTO lesions of at least one major epicardial coronary artery by coronary angiography from January 1st 2019 to 31st December 2019 in Beijing Anzhen Hospital, Capital Medical University. The main exclusion criteria for this study were: ST-elevation myocardial infarction (STEMI), suspected familial hypertriglyceridaemia or current use of triglyceride-lowering medications, type 1 diabetes mellitus, acute infection, severe renal failure (estimated glomerular filtration rate < 30 mL/min), including those receiving renal replacement therapy, alanine aminotransferase or aspartate aminotransferase ≥ 5 times the normal upper limit, left ventricular ejection fraction < 30% or New York Heart Association (NYHA) ≥ Grade III. We also excluded patients with incomplete key information, including triglyceride (TG) and fasting blood glucose (FBG) levels. Patients were classified into categories by TyG index tertiles. The levels of the TyG index in each tertile were as follows: T1: ≤ 8.50, T2: > 8.50, ≤ 8.95, T3: > 8.95. The institutional review board of Beijing Anzhen Hospital of Capital Medical University approved the study design and implementation. The waiver of informed consent was granted and the personal information of enrolled patients was concealed.

### Definitions

The TyG index was calculated by the formula: ln [TG (mg/dl) × glucose (mg/dl)/2] [12]. Diabetes was defined as fasting blood glucose ≥ 7.0 mmol/L, random blood glucose ≥ 11.1 mmol/L, and/or blood glucose ≥ 11.1 mmol/L 2 h after the oral glucose tolerance test or current use of anti-diabetic medication [13]. Hypertension was defined as systolic blood pressure (SBP) ≥ 140 mmHg, diastolic blood pressure (DBP) ≥ 90 mmHg, and/or in-use of antihypertensive drugs [14]. Unstable angina was defined as myocardial ischaemia at rest or during minimal exercise without cardiac enzyme elevation [15]. CTO was defined as a coronary angiographically demonstrated lesion with thrombolysis in myocardial infarction (TIMI) grade 0 blood flow, no thrombus, an unstained proximal fibrous cap, and mature collateral circulation, with a duration of occlusion for more than 3 months [16].

### Perioperative management

All PCI procedures and medical treatments were in accordance with current guidelines on myocardial revascularization [17]. The detailed PCI strategy was determined by experienced cardiologists. Coronary angiogram data were analysed by at least two experienced cardiologists. Multivessel diseases were defined as more than two main epicardial coronary artery stenoses ≥ 50%. Diffuse lesions were referred to as single stenotic lesions ≥ 20 mm in length. Procedurally successful CTO PCI was defined as achievement of TIMI grade 2 or greater antegrade flow in all ≥ 2.5-mm distal branches without any in-hospital major adverse cardiovascular events, including death, acute myocardial infarction, or clinically driven target vessel revascularization.

### Data collection

Demographic, medical history, PCI procedure information and medical treatment at discharge were collected from the medical information recording system. Blood samples were collected in the morning after overnight fasting and tested on the same day using standard laboratory methods in the central laboratory. Patients included in the study were routinely followed up by telephone communication, medical history system, or outpatient service.

### Outcomes

All enrolled patients were followed up for 24 months except when lost to follow-up or if they reached an endpoint of observation. The primary endpoint was the composite of MACCEs, which included all-cause death, nonfatal myocardial infarction, unplanned revascularization and nonfatal ischaemic stroke. Nonfatal stroke was defined as acute cerebral infarction diagnosed by typical symptoms or imaging [18]. Myocardial infarction (MI) was defined according to the fourth general definition of MI [19]. Unplanned repeat revascularization was defined as any accidental PCI or coronary artery bypass grafting (CABG) after indexing procedures.

### Statistical analysis

Continuous variables are presented as the mean ± SD or median (interquartile range). One-way ANOVA was used for the comparison of normally distributed data and the Kruskal‒Wallis test was used for the comparison of nonnormally distributed data. The categorical variables are presented as the number of cases with percentages and were tested by the χ^2^ test (Fisher’s exact test). A Cox proportional hazard regression model was used to analyse the relationship between the TyG index and cardiovascular events and adjusted for multiple models: Model 1: adjusted for age, sex, and body mass index (BMI); Model 2: Model 1 + current smoker, hypertension, diabetes mellitus, dyslipidaemia, previous PCI, and previous CABG; Model 3: Model 2 + acute myocardial infarction (AMI), FBG, total cholesterol (TC), low-density lipoprotein cholesterol (LDL-C), successful CTO treatment, and use of statins, ezetimibe, or antidiabetic medication. A stepwise regression method was used. The survival curve for each group was plotted by using the Kaplan–Meier method and analysed by the log-rank test. RCS analysis was conducted to explore correlations between the TyG index level and cardiovascular risk. Sensitivity analysis was performed according to whether the CTO PCI was procedurally successful and the diabetes status. We used SPSS 26.0 (IBM Corp., Armonk, NY, USA) and R 4.1.3 (R Foundation for Statistical Computing, Vienna, Austria) for data analysis. *P* < 0.05 was considered statistically significant (double tails).

## Results

**Fig. 1:**
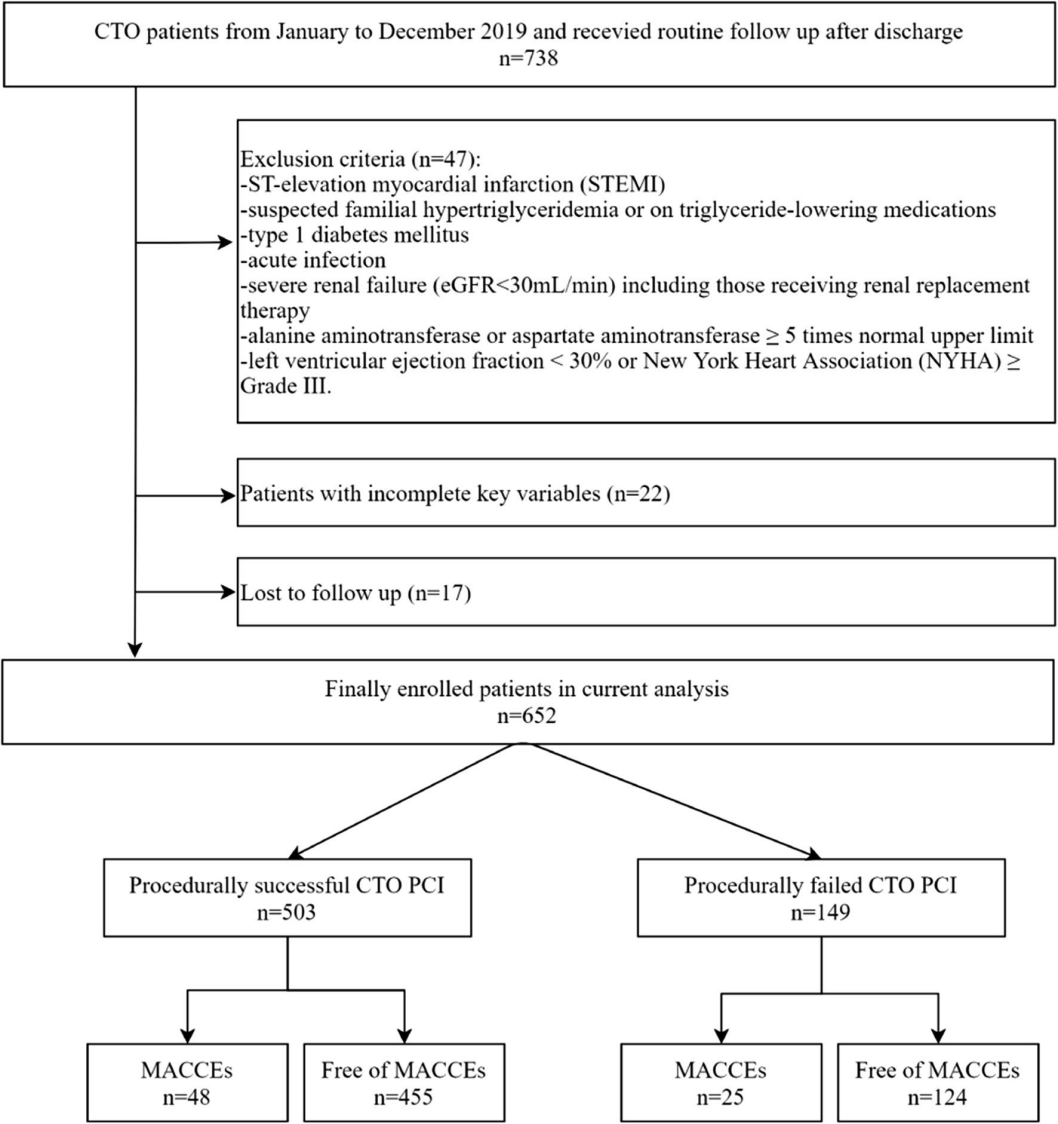
Flow diagram of the study. *CTO* chronic total occlusion, *eGFR* estimated glomerular filtration rate, *MACCEs* major adverse cardiac and cerebrovascular events, *PCI* percutaneous coronary intervention

As shown in Fig. 1, 17 patients were lost during follow-up and 652 patients were enrolled in the final analysis. During the 22.8 ± 3.84-month follow-up period, 73 (11.19%) MACCEs occurred. The average time interval of MACCEs was 13.7 ± 6.15 months.

### Baseline characteristics

The baseline characteristics are shown in Table 1. Male patients comprised 83.7% of the patients in the study, and the mean age of the patients was 58.2 ± 10.49 years. The mean TyG index was 8.8 ± 0.57. Among the 652 patients, procedurally successful CTO PCIs were performed in 503 (77.15%) patients. Patients in the higher TyG index tertiles tended to have hypertension, diabetes and dyslipidaemia; and to higher FBG, TC, TG and LDL-C. Patients with high TyG index levels were more likely to take antidiabetic agents. In terms of angiography results, an increased prevalence of multivessel and diffuse lesions was found as the TyG index increased.

**Table 1:** Baseline characteristics of patients according to TyG index levels

Variable	Total	TyG index level			*P* value
		T1 (≤ 8.5)	T2 (> 8.5, ≤ 8.95)	T3 (> 8.95)	
N (%)	652	217	223	212	-
Age, y	58.2 ± 10.49	59.1 ± 10.78	58.5 ± 10.33	56.9 ± 10.29	0.069
Male, n (%)	546(83.7)	189(87.1)	188(84.3)	169(79.7)	0.113
BMI, kg/m2	26.0 ± 3.19	25.2 ± 3.06	26.2 ± 3.06	26.7 ± 3.30	< 0.001
Heart rate, bpm	72.22 ± 10.60	71.11 ± 10.04	72.43 ± 11.37	73.13 ± 10.26	0.133
SBP, mmHg	128.34 ± 17.32	126.41 ± 17.49	128.02 ± 17.60	130.66 ± 16.66	0.037
Medical history and risk factors, n (%)					
Current smoker	238(36.5)	84(38.7)	75(33.6)	79(37.3)	0.522
Hypertension	431(66.1)	121(55.8)	155(69.5)	155(73.1)	< 0.001
Diabetes	240(36.8)	53(24.4)	71(31.8)	116(54.7)	< 0.001
Dyslipidemia	481(73.8)	153(70.5)	163(73.1)	165(77.8)	0.217
Previous MI	108(16.6)	33(15.2)	39(17.5)	36(17.0)	0.797
Previous Stroke	37(5.7)	14(6.5)	17(7.6)	6(2.8)	0.081
Previous PCI	172(26.4)	61(28.1)	57(25.6)	54(25.5)	0.778
Previous CABG	32(4.9)	9(4.1)	14(6.3)	9(4.2)	0.505
Laboratory Tests					
eGFR, ml/min/1.73m^2^	126.53 ± 32.44	132.30 ± 39.25	122.95 ± 25.59	124.38 ± 30.49	0.005
FBG, mmol/L	6.16 ± 1.36	5.5979 ± 0.93	6.0504 ± 1.03	6.86 ± 1.69	< 0.001
TC, mmol/L	4.01 ± 1.05	3.72 ± 0.98	3.86 ± 0.86	4.45 ± 1.15	< 0.001
TG, mmol/L	1.28(0.93, 1.76)	0.84(0.70, 0.97)	1.30(1.15, 1.50)	2.05(1.74, 2.54)	< 0.001
LDL-C, mmol/L	2.39 ± 0.86	2.21 ± 0.85	2.33 ± 0.75	2.62 ± 0.93	< 0.001
TyG index	8.8 ± 0.57	8.2 ± 0.25	8.7 ± 0.13	9.4 ± 0.43	< 0.001
ACS type, n (%)					
Unstable angina	567(87.0)	185(85.3)	194(87.0)	188(88.7)	0.574
AMI	78(12.0)	29(13.4)	26(11.7)	23(10.8)	0.714
Medication at discharge, n (%)					
Aspirin	640 (98.2)	214(98.6)	219(98.2)	207(97.6)	0.752
clopidogrel	426(65.3)	142(65.4)	147(65.9)	137(64.6)	0.960
ticagrelor	226(34.7)	75(34.6)	76(34.1)	75(35.4)	0.960
statin	639(98.0)	214(98.6)	216(96.9)	209(98.6)	0.321
Ezetimibe	134(20.6)	37(17.1)	44(19.7)	53(25.0)	0.117
Any antidiabetic agents	167(25.6)	31(14.3)	49(22.0)	87(41.0)	< 0.001
Angiographic Coronary anatomy, n (%)					
Any left main disease	150(23.0)	53(24.4)	53(23.8)	44(20.8)	0.629
Multivessel disease	392(60.1)	125(57.6)	121(54.3)	146(68.9)	0.005
Lesions > 20 mm	295(45.2)	81(37.3)	104(35.3)	110(51.9)	0.009
Ostial lesion	70(10.7)	28(12.9)	23(10.3)	19(9.0)	0.406
Bifurcation	20(3.1)	8(3.7)	6(2.7)	6(2.8)	0.808
Treated vessel, n (%)					
LM	61(9.4)	24(11.1)	21(9.4)	16(7.5)	0.458
LAD	415(63.7)	148(68.2)	141(63.2)	126(59.4)	0.166
LCX	264(40.5)	96(44.2)	90(40.4)	78(36.8)	0.291
RCA	316(48.5)	91(41.9)	115(51.6)	110(51.9)	0.062
Mean stent diameters, mm	2.9 ± 0.40	2.8 ± 0.38	2.9 ± 0.38	2.9 ± 0.42	0.490
Total length of stents, mm	50(29,71)	50(28,69.5)	51(29,71)	50(20,74)	0.872
Treatment of CTO lesions, n (%)					
LAD	251(38.5)	92(42.4)	77(34.5)	82(38.7)	0.237
LCX	186(28.5)	72(33.2)	66(29.6)	48(22.6)	0.049
RCA	227(34.8)	57(26.3)	84(37.7)	86(40.6)	0.004
Successful CTO PCI	503(77.1)	169(77.9)	165(74.0)	169(79.7)	0.346
Mean stent diameters at CTO lesion, mm	2.8 ± 0.38	2.8 ± 0.37	2.8 ± 0.38	2.8 ± 0.40	0.340
Total length of stents at CTO lesion, mm	40(26,66)	40(28,65)	38.5(25.25,64.75)	45(28.5,68)	0.768

*TyG index* triglyceride-glucose index, *BMI* body mass index, *SBP* systolic blood pressure, *MI* myocardial infarction, *PCI* percutaneous coronary intervention, *CABG* coronary artery bypass grafting, *eGFR* estimated glomerular filtration rate, *FBG* fasting blood glucose, *HbA1c* glycosylated haemoglobin, *TC* total cholesterol, *TG* triglyceride, *LDL-C* low-intensity lipoprotein-cholesterol, *ACS* acute coronary syndrome, *AMI* acute myocardial infarction, *LM* left main coronary artery, *LAD* left anterior descending artery, *LCX* left circumflex artery, *RCA* right coronary artery, *CTO* chronic total occlusion

### Relationship between the TyG index and cardiovascular events in CTO patients

The relationship between the TyG index and cardiovascular events in CTO patients is shown in Table 2. As the TyG index tertiles increased, the risk of cardiovascular events increased significantly after being adjusted by different models. Model 3 revealed a 2.09-fold risk for MACCEs among those with the highest TyG index compared with those with the lowest TyG index (T2 vs. T1: HR 1.24, 95% CI 0.65–2.38, *P* = 0.057; T3 vs. T1: HR 2.09, 95% CI 1.14–3.86, *P* = 0.018; *P* for trend = 0.036).

**Table 2:** Relationship between TyG index and cardiovascular events in CTO patients

TyG Tertiles	HR (95% CI)			
	Unadjusted	Model 1^a^	Model 2^a^	Model 3^a^
T1	1 (reference)^#^	1 (reference)^#^	1 (reference)^#^	1 (reference)^#^
T2	1.29 (0.67–2.47)	1.29 (0.67–2.47)	1.32 (0.69–2.52)	1.24 (0.65–2.38)
T3	2.44 (1.35–4.39)	2.44 (1.35–4.39)	2.46 (1.37–4.44)	2.09 (1.14–3.86)

^a^The log-rank test and backward stepwise selection methods in a Cox proportional hazards regression model were performed; Model 1: adjusted for age, sex, BMI; Model 2: Model 1 + current smoker, hypertension, diabetes mellitus, dyslipidemia, previous PCI, previous CABG; Model 3: Model 2 + acute myocardial infarction, FBG, TC, LDL-C, successful CTO treatment, using of statin, ezetimibe, or antidiabetic medication. TyG index tertiles: T1: ≤ 8.50, T2: > 8.50, ≤ 8.95, T3: > 8.95

^#^*P* < 0.05

**Fig. 2:**
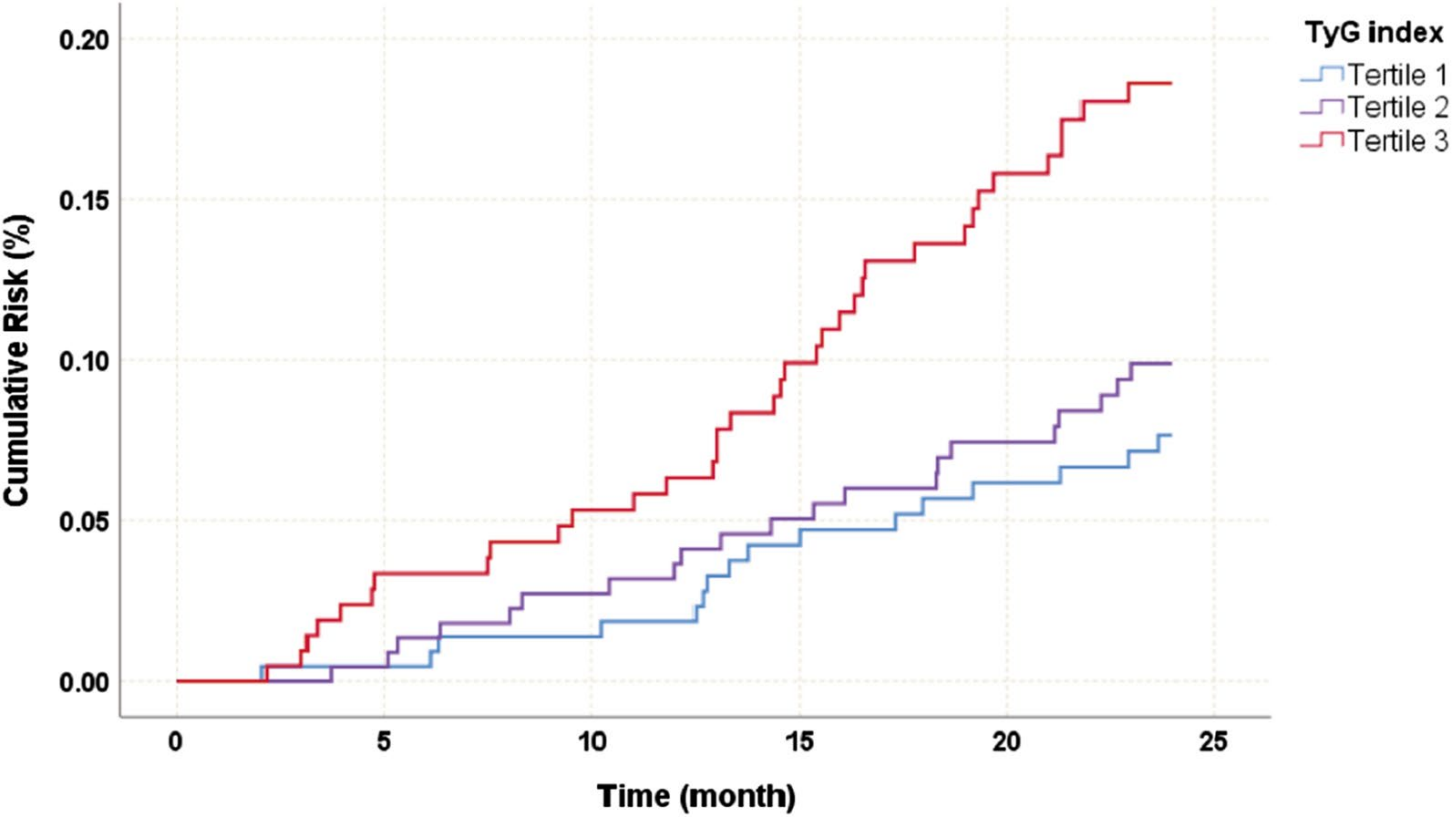
Kaplan–Meier curve of cumulative risk of cardiovascular events according to different TyG index levels. *TyG index* triglyceride-glucose index

The Kaplan‒Meier curve was used to show the cumulative risk of cardiovascular events by TyG index tertiles (Fig. 2). As TyG index tertiles increased, the cumulative risk of cardiovascular events became significantly higher (log-rank *P* = 0.004).

**Fig. 3:**
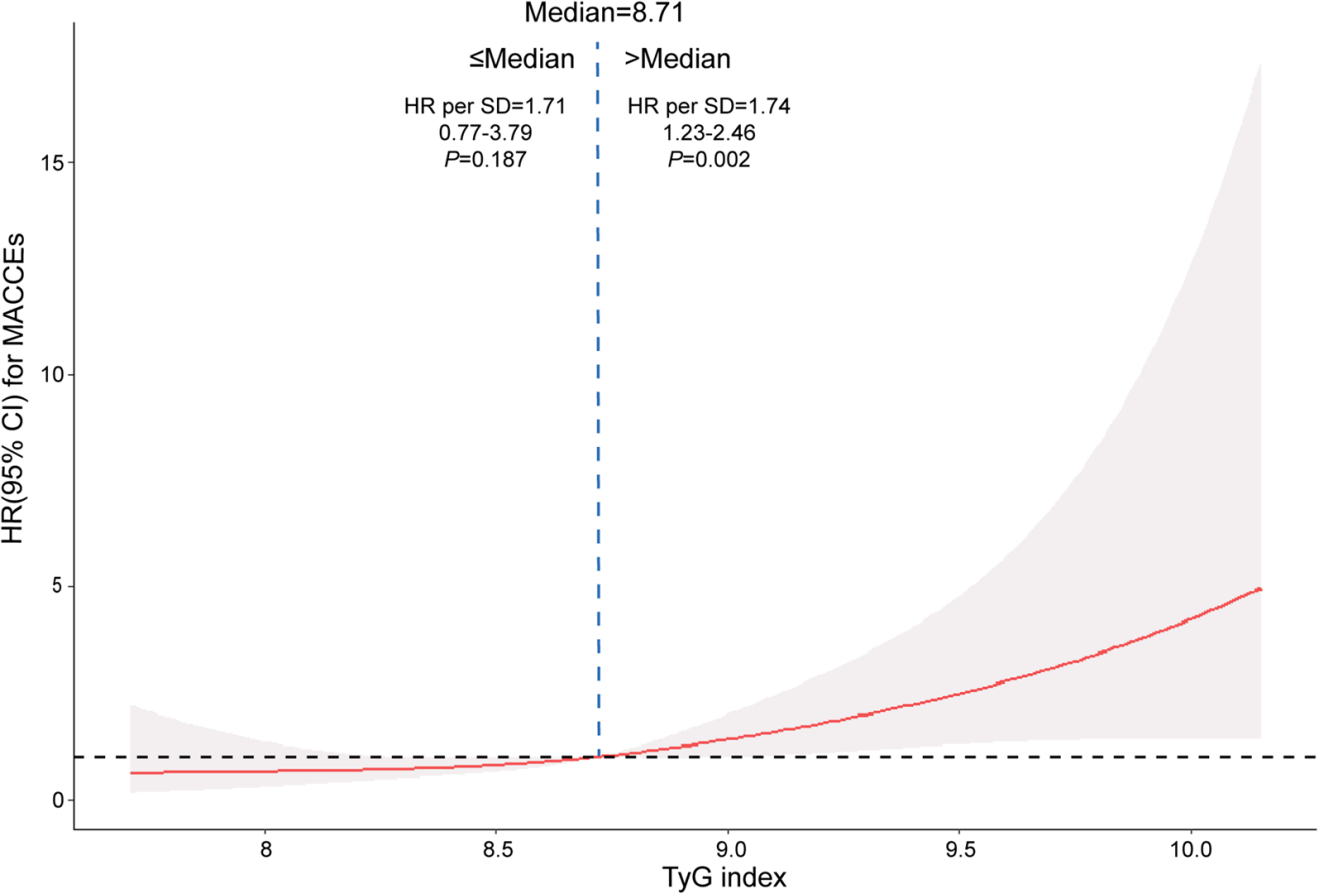
The restricted cubic spline of MACCEs and the TyG index. *HR* hazard ratio, *CI* confidence interval, *MACCEs* major adverse cardiovascular and cerebral events, *TyG index* triglyceride-glucose index

To visualize the relationship between the TyG index and cardiovascular risk, we fitted a RCS model (Fig. 3). The RCS curve appears to be J-shaped, with the HR for MACCEs significantly increasing as the TyG index increased over 8.71 (HR per SD 1.74, 95% CI 1.23–2.46, *P* = 0.002). For a TyG index ≤ 8.71, the HR per SD was 1.709 (95% CI 0.77–3.79, *P* = 0.187).

We performed sensitivity analyses in this study. First, we tested the effect of successful CTO PCI on the association between the TyG index and incident MACCEs. As shown in Table 3, the prognostic value of the TyG index in successful CTO PCI patients was similar to the findings of the main analysis (T2 vs. T1: HR 1.33, 95% CI 0.58–3.04, *P* = 0.495; T3 vs. T1: HR 2.69, 95% CI 1.29–5.60, *P* = 0.008, *P* for trend = 0.013), but not in patients with failed CTO PCI. Second, we stratified the patients by diabetes status. We found that high TyG index tertile was related to cardiovascular risk in nondiabetes patients (T2 vs. T1: HR 1.01, 95% CI 0.41–2.51, *P* = 0.979; T3 vs. T1: HR 2.35, 95% CI 1.06–5.20, *P* = 0.036, *P* for trend = 0.040), but not in diabetes patients.

**Table 3:** Sensitivity analysis of the relationship between the TyG index and cardiovascular events stratified by diabetes status and successful or failed CTO PCI

	T1		T2		T3	
	HR (95%CI)	*P* value	HR (95%CI)	*P* value	HR (95%CI)	*P* value
DM^#^	1 (reference)	0.356	1.81 (0.68–4.86)	0.237	2.11 (0.74–5.98)	0.162
Non-DM^#^	1 (reference)	0.040	1.01 (0.41–2.51)	0.979	2.35 (1.06–5.20)	0.036
Successful CTO PCI^*^	1 (reference)	0.013	1.33 (0.58–0.04)	0.495	2.69 (1.29–5.60)	0.008
Failed CTO PCI^*^	1 (reference)	0.373	0.52 (0.15–1.79)	0.299	1.32 (0.37–4.67)	0.672

^#^Adjusted for age, sex, BMI, current smoker, hypertension, dyslipidemia, previous PCI, previous CABG, AMI, FBG, TC, LDL-C, using of statin, ezetimibe, or antidiabetic medication, successful CTO PCI. TyG index Tertiles in diabetes mellitus patients: T1: ≤ 8.65, T2: > 8.65, ≤ 9.26, T3: > 9.26; TyG index Tertiles in non-diabetes mellitus patients: T1: ≤ 8.43, T2: > 8.43, ≤ 8.83, T3: > 8.83

^*^Adjusted for age, sex, BMI, current smoker, hypertension, DM, dyslipidemia, previous PCI, previous CABG, AMI, FBG, TC, LDL-C, using of statin, ezetimibe, or antidiabetic medication. TyG index Tertiles in successful CTO PCI patients: T1: ≤ 8.50, T2: > 8.50, ≤ 8.96, T3: > 8.96; TyG index Tertiles in failed CTO PCI patients: T1: ≤ 8.53, T2: > 8.53, ≤ 8.91, T3: > 8.91

## Discussion

In this study, we demonstrated the correlation between the TyG index and the risk of cardiovascular events in patients with CTO lesions for the first time. The risk of cardiovascular adverse events in CTO patients increased significantly with the increase in the TyG index. A higher TyG index (> 8.71) was associated with significant increases per SD of the TyG index, and no such significant association was found for a lower TyG index (≤ 8.71). The findings of our study extend our understanding of TyG as a tool for cardiovascular events, especially for patients with CTO lesions. This suggests that the TyG index may be helpful in identifying more patients at high risk who need more aggressive treatment and follow‐up strategies.

Our observations that an elevated TyG index was associated with a increased cardiovascular risk are consistent with several recent studies. Among patients with different pathological processes of atherosclerosis, including arterial stiffness, coronary artery calcification, in-stent restenosis and STEMI, ACS, and nonobstructive MI, the TyG index appears to be effective in predicting cardiovascular events [10, 20–22]. However, few researchers have studied the relationship between the TyG index and cardiovascular risk in CTO patients. A study has shown the an elevated TyG index is closely related to the underdevelopment of collateral circulation in CTO lesions, and its risk assessment performance is better than that of a single metabolic abnormality index [23]. Similar to the current study, HOMA-IR, an index of IR, has been reported to predict cardiovascular events in patients with non-ST-elevation myocardial infarction (NSTEMI) combined with type 2 diabetes mellitus (T2DM) and CTO lesions, but the sample size was small and highly selective [24]. Our findings are consistent with these studies.

Insulin resistance, which is represented by the TyG index, is correlated with CTO. Animal research has proven that IR can affect the expression of vascular endothelial growth factor (VEGF) and its receptors, which are major angiogenic factors expressed in response to hypoxia [25]. Insufficient response of the myocardium to angiogenesis under ischaemia may lead to poor collateral circulation. In addition, increased production of reactive oxygen species (ROS) and persistent endothelial dysfunction are also the potential mechanisms of the poor collateral development in patients with IR [26, 27]. Besides, when insulin resistance occurs in adipose tissue, the level of circulating free fatty acids (FFAs) increases. A high concentration of FFAs increases TG-rich very-low-density lipoprotein (VLDL), which leads to the clearance of high-density lipoprotein (HDL) and reduces its concentration [28, 29]. All of these changes contributed to atherogenic dyslipidaemia and plague progression caused by insulin resistance. A clinical study demonstrated that IR is associated with poor collateral vessel formation [30]. A high TyG index is also reported to be strongly associated with poor coronary collateralization in CTO patients [23]. However, well-developed collateral circulation of the coronary artery can improve the survival and prognosis of patients with coronary artery disease [31, 32]. These results indicated that IR, which is represented by the TyG index, may play a key role in the development of collateral circulation and could explain why a high TyG index could predict cardiovascular events in CTO patients.

In the sensitivity analysis, the TyG index appeared to be significantly correlated with cardiovascular risk only in patients who received successful CTO PCI but not in patients whose CTO lesion was not opened successfully. We speculated that several reasons might be responsible for this. In our opinion, the main reason for the result of the sensitivity analysis was the insufficient sample size of the failed CTO PCI subgroup. In addition, stents were implanted in most of the patients with successful CTO PCI but not in patients with failed PCI. Studies have shown that the TyG index appears to be associated with cardiovascular events in patients who undergo PCI [12, 33]. Furthermore, failed PCI may have damaged a significant amount of collateral circulation during the opening of the occluded lesion, and this may obscure the relationship between the TyG index and collateral circulation, as shown in a previous study [23]. Similarly, the TyG index only appeared to be associated with cardiovascular risk in nondiabetes patients. Some previous studies have indicated that the TyG index could predict cardiovascular events effectively both in patients with and without DM [33–35], but a study has found similar associations in diabetic populations but not in nondiabetic populations [36]. In this study, we assumed that this was mainly because the insufficient sample size in the DM group led to decreased test efficiency. To resolve this problem, larger randomized controlled trials are needed in the future to verify this study.

The current study has several limitations that need to be considered. First, the sample size and qualities were limited because of the single-centre retrospective study design. Second, we only included the baseline of the TyG index and the change in the TyG index in the follow-up period was missed, which may have an impact on the prognosis [37]. Third, although we adjusted the models according to the use of antilipemic and antidiabetic drugs, the results may be biased because we did not account for the type, intensity, and changes of these drugs. Finally, the present results may not be applicable to other ethnic groups because the participants in our study were only Chinese.

## Conclusion

The study indicated that the TyG index has significant relevance to cardiovascular risk in CTO patients and suggested that the TyG index may be a reliable tool to predict cardiovascular events in CTO patients. The findings of our study extend our understanding of the TyG index as a tool for cardiovascular events, especially for patients with CTO lesions.

## Data Availability

The data used in the current study are available on reasonable request from the corresponding author.

## Abbreviations

ACS: Acute coronary syndrome
AMI: Acute myocardial infarction
BMI: Body mass index
CABG: Coronary artery bypass grafting
CI: Confidence interval
CTO: Chronic total occlusion
DBP: Diastolic blood pressure
DM: Diabetes mellitus
FBG: Fasting blood glucose
FFA: Free fatty acids
HDL: High-density lipoprotein
HIEC: Hyper insulinemic-euglycemic glucose clamp
HR: Hazard ratio
IR: Insulin resistance
LAD: Left anterior descending artery
LCX: Left circumflex artery
LDL-C: Low-density lipoprotein cholesterol
LM: Left main coronary artery
MACCEs: Major adverse cardiovascular and cerebral events
MI: Myocardial infarction
NSTEMI: Non-ST-elevation myocardial infarction
NYHA: New York Heart Association
PCI: Percutaneous coronary intervention
RCA: Right coronary artery
RCS: Restricted cubic spline
ROS: Reactive oxygen species
SBP: Systolic blood pressure
SD: Standard deviation
STEMI: ST-elevation myocardial infarction
T2DM: Type 2 diabetes mellitus
TC: Total cholesterol
TG: Triglyceride
TIMI: Thrombolysis in myocardial infarction
TyG index: Triglyceride-glucose index
VEGF: Vascular endothelial growth factor
VLDL: Very-low-density lipoprotein
